# Immunomodulation by *Bifidobacterium infantis* 35624 in the Murine Lamina Propria Requires Retinoic Acid-Dependent and Independent Mechanisms

**DOI:** 10.1371/journal.pone.0062617

**Published:** 2013-05-21

**Authors:** Patrycja Konieczna, Ruth Ferstl, Mario Ziegler, Remo Frei, Dirk Nehrbass, Roger P. Lauener, Cezmi A. Akdis, Liam O'Mahony

**Affiliations:** 1 Swiss Institute of Allergy and Asthma Research (SIAF), University of Zurich, Davos, Switzerland; 2 Christine Kühne-Center for Allergy Research and Education (CK-CARE), Davos, Switzerland; 3 AO Research Institute Davos (ARI), Davos, Switzerland; 4 Hochgebirgsklinik Davos-Wolfgang, Davos, Switzerland; Charité-University Medicine Berlin, Germany

## Abstract

Appropriate dendritic cell processing of the microbiota promotes intestinal homeostasis and protects against aberrant inflammatory responses. Mucosal CD103^+^ dendritic cells are able to produce retinoic acid from retinal, however their role *in vivo* and how they are influenced by specific microbial species has been poorly described. *Bifidobacterium infantis* 35624 (*B. infantis)* feeding to mice resulted in increased numbers of CD103^+^retinaldehyde dehydrogenase (RALDH)^+^ dendritic cells within the lamina propria (LP). Foxp3^+^ lymphocytes were also increased in the LP, while T_H_1 and T_H_17 subsets were decreased. 3,7-dimethyl-2,6-octadienal (citral) treatment of mice blocked the increase in CD103^+^RALDH^+^ dendritic cells and the decrease in T_H_1 and T_H_17 lymphocytes, but not the increase in Foxp3^+^ lymphocytes. *B. infantis* reduced the severity of DSS-induced colitis, associated with decreased T_H_1 and T_H_17 cells within the LP. Citral treatment confirmed that these effects were RALDH mediated. RALDH^+^ dendritic cells decreased within the LP of control inflamed animals, while RALDH^+^ dendritic cells numbers were maintained in the LP of *B. infantis*-fed mice. Thus, CD103^+^RALDH^+^ LP dendritic cells are important cellular targets for microbiota-associated effects on mucosal immunoregulation.

## Introduction

The mammalian gastrointestinal microbiota is required for optimal host development and ongoing immune homeostasis [1]–[3]. The microbiota aids in the digestion of foods, competes with pathogens, degrades mucin and promotes the differentiation of epithelial cells and mucosa-associated lymphoid tissue. In addition, the composition and metabolic activity of the microbiota has profound effects on proinflammatory activity and the induction of immune tolerance by influencing a broad range of mucosal cell types including epithelial cells, dendritic cells, iNKT cells and T lymphocyte subset activity [4]–[6].

Gastrointestinal immune homeostasis is dependent on a number of local conditioning factors that reduce pathological proinflammatory responses to non-pathogenic microbes. For example, epithelial-derived cytokines such as TSLP and IL-25 limit dendritic-cell secretion of IL-12 and IL-23, while promoting IL-10 secretion [7]. In addition, certain dendritic cell subsets within the mucosa can metabolize vitamin A into retinoic acid, such as the CD103^+^ dendritic cell subset [8], [9]. Retinoic acid is synthesized from stored or dietary retinol by the oxidation of retinol to retinal, followed by oxidation of retinal to retinoic acid. The final step is catalyzed by aldehyde dehydrogenase family 1, subfamily A1 (Aldh1a1) and ALDH1 subfamily A2 (Aldh1a2), also called RALDH enzymes. 3,7-dimethyl-2,6-octadienal (citral) blocks RALDH enzymatic activity. Dendritic cell-derived retinoic acid has dramatic effects on dendritic cell activity and lymphocyte subset plasticity. Retinoic acid can have seemingly conflicting effects on lymphocyte polarization, such as promoting T_H_17 cells or T_reg_ cells [10]. The promotion of T_H_17 versus T_reg_ phenotypes may be related to the local concentration of retinoic acid, the dendritic cell subset secreting retinoic acid, the local level of pro-inflammatory mediators and TGF-β, concomitant toll-like receptor activation or induction of specific microRNA [11]–[14]. So far, the role of specific microbial species in influencing retinoic acid metabolism and CD103^+^RALDH^+^ dendritic cells *in vivo* has been poorly understood.

*Bifidobacterium longum* subsp. *infantis* 35624 (*B. infantis*) was originally isolated from resected human healthy gastrointestinal tissue and human clinical studies have demonstrated its efficacy in Irritable Bowel Syndrome patients [15], [16]. In addition, murine studies have demonstrated that this microbe protects against inflammatory disorders across a range of inflammatory conditions including colitis, pathogen infection, arthritis and respiratory inflammation [17]–[20]. Previously, *in vitro* studies with human dendritic cells suggested that promotion of retinoic acid metabolism by *B. infantis* was a key regulatory feature of this bacterium [21]. In this report, we demonstrate that *B. infantis* feeding to mice results in increased CD103^+^RALDH^+^ dendritic cells within the mucosa, which are responsible for the suppression of T_H_1 and T_H_17 lymphocytes and amelioration of dextran sulfate sodium (DSS)-induced colitis.

## Methods

### Bacteria and animal models

Wild-type C57BL/6 mice were obtained from Charles River and maintained under specific pathogen free conditions. Mice were housed at the AO Research Institute, Davos, Switzerland, in individually ventilated cages for the duration of the study, and all experimental procedures were carried out in accordance with Swiss law. Experimental protocols were approved by the Ethics Committee of the “Amt für Lebensmittelsicherheit und Tiergesundheit Graubünden”, application number 2011–15. In the first experiment, three groups of mice were utilized (n = 8 per group). Group 1 did not receive any bacterial supplementation, while groups 2 and 3 were fed *B. infantis* for 7 days. Each day lyophilized bacteria were resuspended in sterile water to final concentration of 6×10^8^ colony forming units (cfu)/ml. For group 3, 2 mg of citral (Sigma, St. Louis, USA) was dissolved in 10% DMSO (Sigma) and was injected i.p. daily in order to suppress retinoic acid metabolism.

In the dextran sodium sulfate (DSS) colitis model, five groups of wild-type C57BL/6 mice (n = 8 per group) were utilized. Group 1 was the negative control group, which did not receive *B. infantis* and were not administered DSS. Group 2 was the positive control group as these mice received DSS but not *B. infantis*. Groups 3 and 4 were both administered DSS and *B. infantis*, while group 4 was also injected i.p. with citral (as described above). Group 5 received DSS and citral. Mice were fed *B. infantis* for 7 days before colitis induction. Mice received DSS (TdB Consultancy AB, Uppsala) in water (2.5%) for 6 days followed by 2 days without DSS. During this period bacteria were administrated daily by gavage (1×10^9^ cfu/mouse). All mice were euthanized on the final day of the study using cervical dislocation, which was performed by an experienced investigator.

### Cell isolation

Single cell suspensions from mesenteric lymph nodes (MLN) and Peyer's patches (PP) were isolated using C tubes and GentleMACS Dissociator (Miltenyi Biotec, Bergisch Gladbach, Germany) according to manufacturer instructions. LP cells were isolated from the upper part of small intestine (SI). A 5 cm long piece of SI was washed out with cold calcium and magnesium free PBS (CMF-PBS) containing 1 mmol dithiothreitol (DTT) and cold CMF-PBS containing 12 mmol EDTA. The SI was cut into pieces and vortexed in CMF-PBS containing 0.3 mmol EDTA. After centrifugation (300 g/5 minutes) tissue was digested for 45 minutes at 37C in RPMI containing 25 kU/l collagenase IV (Sigma), 150 mg/l DNase I (Roche, Rotkreuz, Switzerland) and 5% fetal calf serum (FCS, Sigma). Cell suspensions were filtered through 70 µm cell strainers, centrifuged (700 g/8 minutes) and washed with CMF-PBS containing 5% FCS, 5 mg/l DNase I, 5 mmol/l EDTA. Finally pellets were resuspended in cRPMI (Invitrogen, LuBioScience, Luzern, Switzerland).

### Flow cytometry and cell imaging

Anti-mouse CD11b, CD11c, MHCII, CD3, CD19 and CD103 antibodies (Biolegend, Lucerna-Chem, Luzerna, Switzerland) were used for characterization of dendritic cell phenotypes. RALDH activity was measured with the ALDEFLOUR kit (Aldagen, Durham, USA) according to manufacturer instructions. Anti-mouse CD3, CD4, CD25, LPAM-1 (integrin α4β7) and CCR9 antibodies (Biolegend) and anti-mouse Foxp3, IL-10, IL-4, IL-17A, IFNγ antibodies (eBioscience San Diego, CA, USA) were used to characterize lymphocyte phenotypes. Cells for intracellular cytokine staining were pre-stimulated for 4 hours with PMA (50 ng/ml, Sigma) and ionomycin (500 ng/ml, Sigma) in the presence of Brefeldin A (1 µg/ml, eBioscience). Flow cytometric analysis was performed using a 10 colour Galios flow cytometer (Beckman Coulter, Brea, USA). Kaluza (Beckman Coulter) was used for data analysis.

MLN and LP cells were incubated *in vitro* for 1 hour with CFSE labeled *B. infantis*. Cells were stained with anti-mouse CD11c and CD103. Bacteria binding was visualized using multispectral imaging flow cytometer Image Stream X (Amnis Corporation, Seattle, USA) and images were analyzed using IDEAS software (Amnis Corporation).

### Histology

Colons were removed, flushed with PBS and wrapped around to generate a swiss roll. Swiss rolls were fixed in 4% paraformaldehye for 12 hours and stored in PBS until paraffin embedding. Following embedding, 3 µm thick sections were stained with Gill's hematoxylin and eosin (Sigma). Mounting was done with Eukitt ® quick-hardening mounting medium (Sigma). Tissue samples were analyzed by a pathologist in a blinded manner. The histology score included assessment of crypts dilatation, inflammatory cells infiltration in LP, inflammatory cells infiltration in submucosa, necrosis of epithelium and submucosal edema. Each parameter was scored from 0 to 5 resulting in a maximum score of 25.

### Myeloperoxidase (MPO) activity test

Pieces of colon were homogenized in 50 mM potassium phosphate buffer, pH 6.0, containing 0.3% hexadecyltrimethyl-ammonium bromide (HTAB, Sigma). Tissue was sonicated on ice for 15 seconds followed by 3 freeze-thaw cycles. Samples were centrifuged and 20 µl of the supernatant was mixed with 200 µl freshly prepared 50 mM potassium phosphate buffer, pH 6.0, containing 0.3% HTAB, O-dianisdine dihydrochloride (0.167 mg/ml, Sigma) and 0.5% hydrogen peroxide (Sigma). Change in absorbance was measured at 450 nm over 4 min. by an ELISA plate reader.

### Cytokine assay

1×10^6^ SI-LP cells were cultured in 1 ml cRPMI containing amphotericin B (6,25 µg/ml, Sigma) and gantamycin (12,5 µg/ml, Sigma) and supernatants were collected after 24 hours. Cytokine secretion was examined by Bio-Plex multiplex suspension array (Bio-Rad Laboratories, Hercules, USA).

### Statistics

Unpaired student t-tests were used to analyse data with a normal distribution, while the non-parametric Mann-Whitney test was used to analyze the non-parametric data. All data analysis was carried out using GraphPad Prism software. A p value of <0.05 was used as the cutoff for statistical significance.

## Results

### *B. infantis* is sampled by Peyer's patch and lamina propria dendritic cells

**Figure 1:**
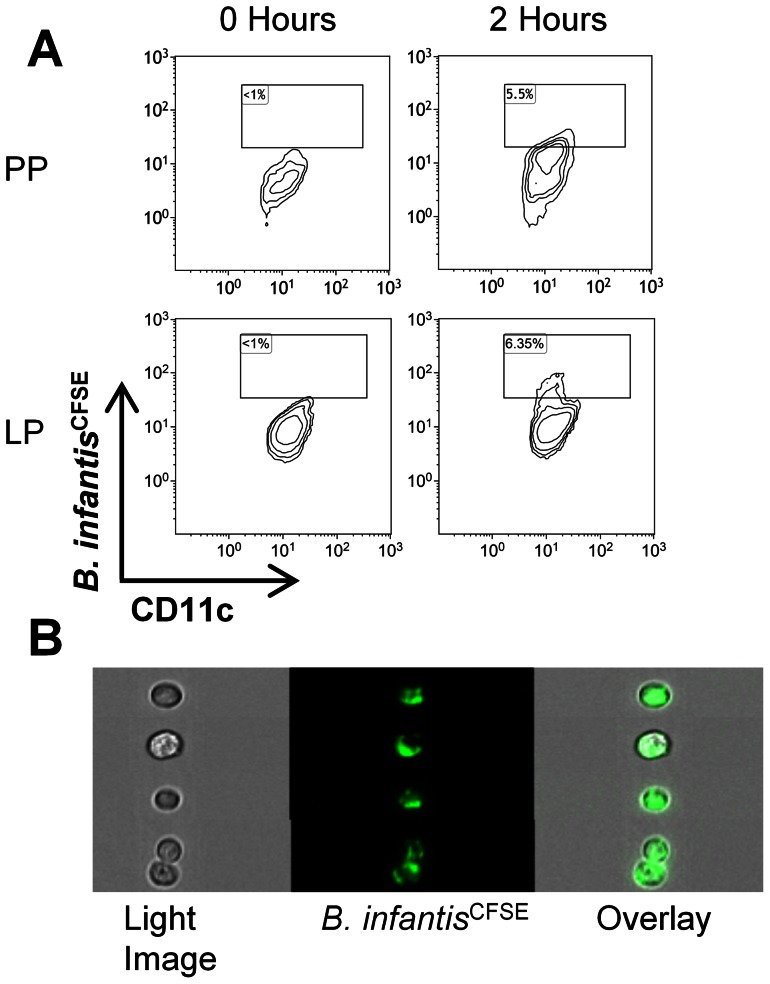
*B.* *infantis* is sampled by dendritic cells within PP and LP. (**a**) Flow cytometric analysis of CD11c^+^MHCII^+^ dendritic cells within the PP and LP at 0 or 2 hours following gavage of CFSE-labelled *B. infantis*, revealed that a subpopulation of dendritic cells at both sites become CFSE^+^. (**b**) Visualization by multispectral flow cytometry imaging confirmed the presence of CFSE-labelled bacteria on PP dendritic cells at 2 hours.

Dendritic cells within the LP and Peyer's patches (PP) have been previously described to sample bacteria from the gastrointestinal lumen. In order to determine whether *B. infantis* was sampled by dendritic cells from either site, CFSE-labelled bacteria were gavaged to mice and single cell suspensions were generated from ileal LP and PP after 2 hours. Within the PP, CD11c^+^MHCII^+^ dendritic cells were identified, which had become CFSE positive 2 hours after feeding (**Figure 1a**). CD11c^+^MHCII^+^ dendritic cells within the LP also became CFSE positive at 2 hours and with higher frequency (**Figure 1a**). The presence of CFSE-labelled bacteria attached to, or internalized by, PP dendritic cells at 2 hours was confirmed using multispectral flow cytometry imaging (**Figure 1b**).

### *B. infantis* induces CD103+ retinoic acid secreting dendritic cells

**Figure 2:**
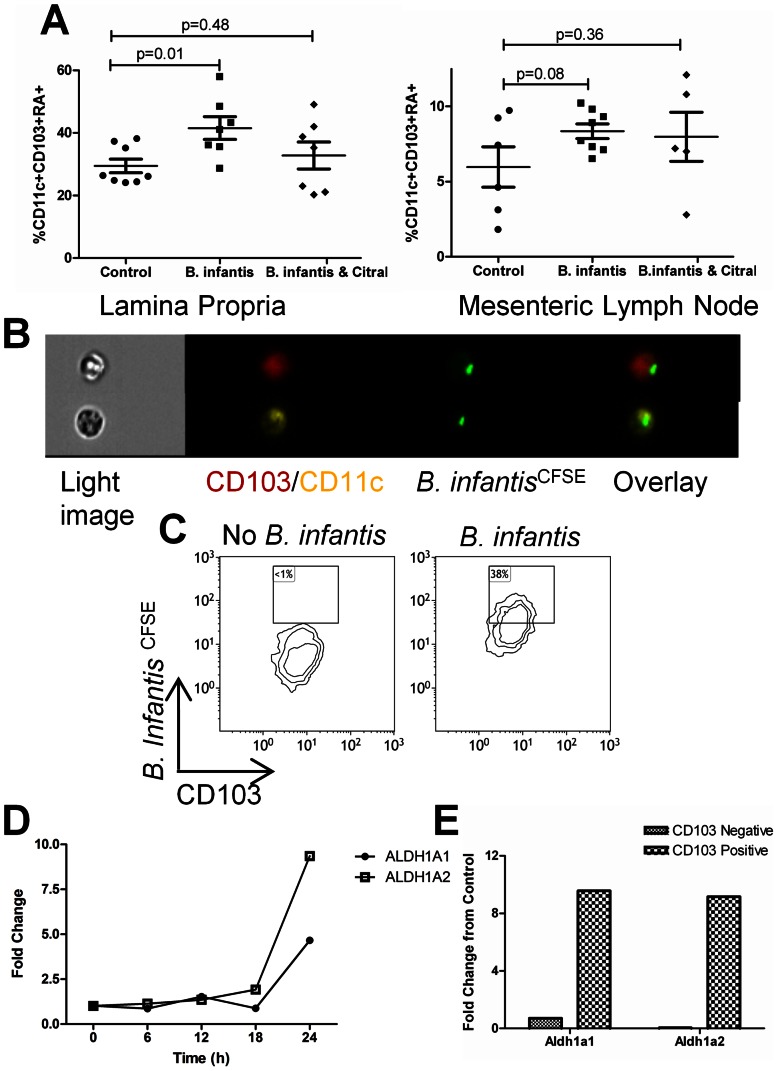
CD103^+^RALDH^+^ dendritic cells are elevated in the LP following *B. infantis* feeding. Flow cytometric assessment of LP and MLN revealed that *B. infantis* feeding is associated with increased CD103^+^RALDH^+^ dendritic cells within the LP (n = 7), compared to the control group (n = 8), analysed using unpaired student t-tests (**a**). Citral blocked the increase in LP CD103^+^RALDH^+^ dendritic cells (n = 7). (**b**) Multispectral flow cytometry imaging identified CD103^+^ dendritic cells that efficiently bind CFSE-labelled *B. infantis*. (**c**) Flow cytometric analysis of CD11c^+^MHCII^+^CD103^+^ dendritic cells from the mucosa demonstrated that approximately 38% of CD103^+^ dendritic cells bound *B. infantis*. Isolated mucosal CD11c^+^ dendritic cells upregulate mRNA for RALDH enzymes following *in vitro* incubation with *B. infantis* (**d**), while the increase in gene expression is specific to CD103^+^ dendritic cells (**e**).

We examined CD103 expression and retinoic acid secretion by dendritic cells in mesenteric lymph nodes (MLN) and ileal LP following *B. infantis* feeding for 7 days. *B. infantis* feeding was associated with a significant increase in the percentage of dendritic cells that were CD103^+^ and metabolizing retinoic acid in the LP, while the increase in retinoic acid metabolizing CD103^+^ dendritic cells in the MLN approached statistical significance (**Figure 2a**). Citral treatment, which blocks retinoic acid metabolism, reduced the *B. infantis*-induced increase in CD103^+^ and retinoic acid metabolizing dendritic cells within the LP. In order to determine if the increase in retinoic acid metabolism was a direct effect of *B. infantis* binding to CD103^+^ dendritic cells, *in vitro* co-incubation with mucosal dendritic cells demonstrated that CD103^+^ dendritic cells were able to bind *B. infantis* at a high frequency, while CD103- dendritic cells bound *B. infantis* at a low frequency (**Figure 2b** **and Figure S1**). Moreover, CD103^+^ dendritic cells gene expression of the retinoic acid metabolizing enzymes *ALDH1a1* and *ALDH1a2* were significantly upregulated following co-incubation with *B. infantis* (**Figure 2d**). In addition, CD103^+^ and CD103^−^ dendritic cells were isolated from the mucosa by flow cytometric sorting and were co-incubated with *B. infantis*. Gene expression for *ALDH1a1* and *ALDH1a2* was significantly increased following *B. infantis* co-incubation only in CD103^+^ dendritic cells, but not CD103^−^ dendritic cells (**Figure 2e**).

### *B. infantis* suppression of T_H_17 cells is retinoic acid-dependent

**Figure 3:**
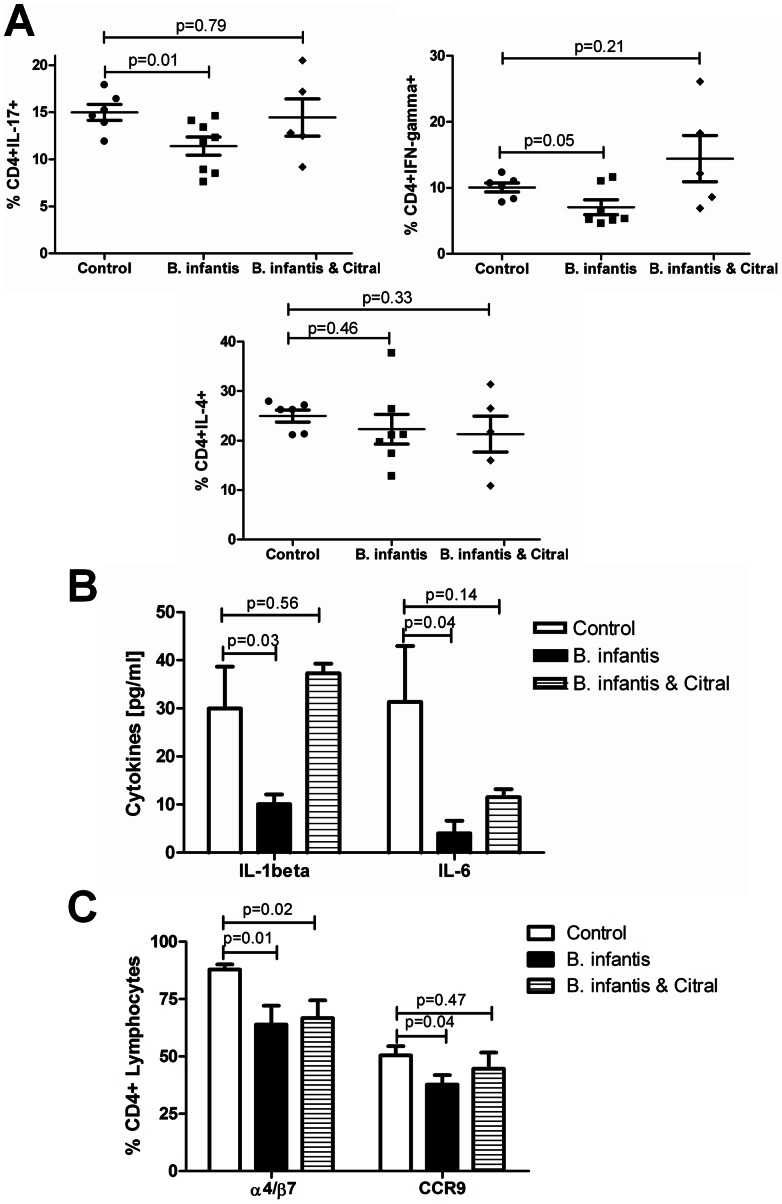
*B. infantis* alters lymphocyte phenotypes within the LP. (**a**) IL-17^+^ lymphocytes were significantly reduced and IFN-gamma^+^ cells were substantially reduced within the LP of *B. infantis*-fed mice (n = 8), compared to the control group (n = 6), an effect that was blocked by citral (n = 5). No effect was observed for IL-4^+^ lymphocytes. (**b**) Isolated LP was cultured *in vitro* and cytokine secretion measured after 24 hours. *B. infantis* feeding reduced the *in vitro* secretion of the T_H_17-polarising cytokines IL-1β and IL-6, which was partially reversed by citral. (**c**) *B. infantis* feeding was associated with a decrease in the proportion of LP lymphocytes expressing the gut homing receptors α4β7 and CCR9. Citral did not reverse the decrease in α4β7^+^ lymphocytes and had a minor influence on CCR9^+^ lymphocytes. Statisical significance was estimated using unpaired student t-tests.

Ileal LP single cell suspensions were examined for IL-17, IFN-gamma and IL-4 positive lymphocytes. The percentage of IL-17^+^ lymphocytes within the LP was significantly reduced following *B. infantis* feeding for 7 days, while the reduction in IFN-gamma^+^ lymphocytes approached statistical significance (**Figure 3a**). Citral treatment reversed the *B. infantis* suppression of IL-17^+^ and IFN-gamma^+^ lymphocytes within the LP. Neither *B. infantis* feeding nor citral treatment altered the percentage of IL-4^+^ lymphocytes within the LP (**Figure 3a**). Isolated LP cells were cultured *in vitro* for 24 hours and spontaneous secretion of T_H_17 polarizing cytokines were measured in culture supernatants. Both IL-1β and IL-6 secretion were significantly reduced by *B. infantis* feeding. Citral reversed the suppression of IL-1β secretion and partially reversed the suppression of IL-6 secretion (**Figure 3b**). One potential explanation for the *B. infantis* suppression of T_H_17 cells within the LP is that *B. infantis* may alter lymphocyte recruitment to the LP. Expression of the intergrin α4β7 and the chemokine receptor CCR9 influence the gut homing of lymphocytes. Both α4β7^+^ and CCR9^+^ lymphocytes populations were significantly suppressed following *B. infantis* feeding (**Figure 3c**). However, citral treatment had no effect on the *B. infantis* suppression of α4β7^+^ lymphocytes, with a minor effect on CCR9^+^ lymphocytes, suggesting that the *B. infantis* effect on lymphocyte homing is independent of retinoic acid metabolism.

### *B. infantis* induction of Foxp3^+^ lymphocytes is retinoic acid-independent

**Figure 4:**
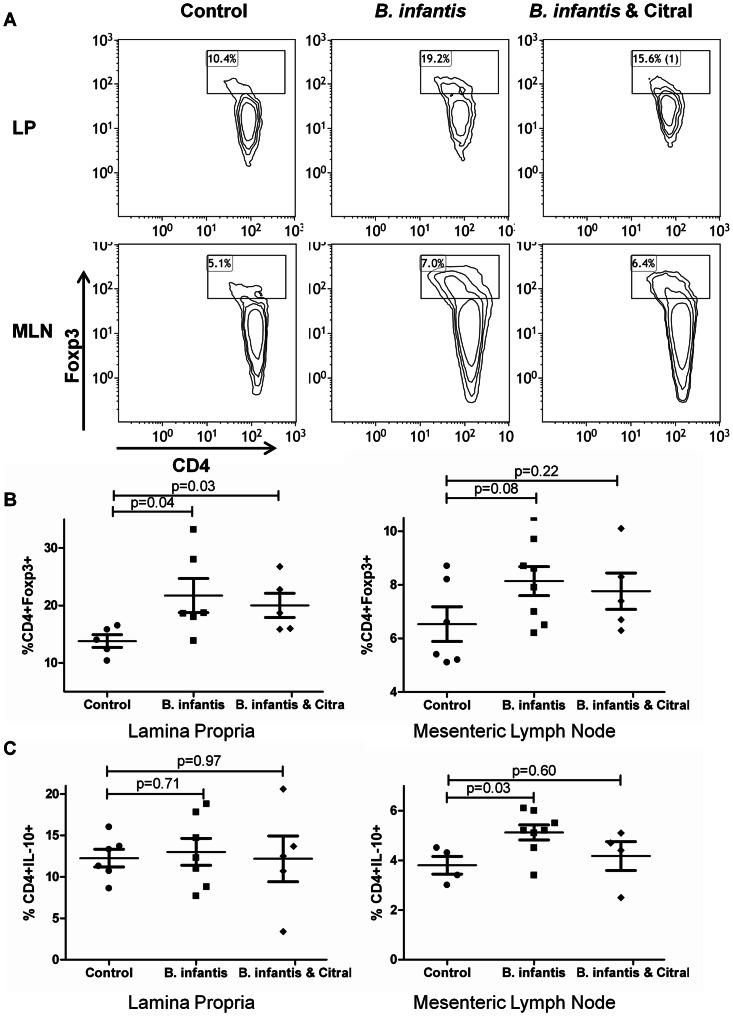
*B. infantis* induction of Foxp3^+^ lymphocytes is not RALDH-dependent. (**a**) Representative flow cytometric dot-plots are illustrated for CD4 and Foxp3 populations within MLN and LP. (**b**) The increase in CD4^+^Foxp3^+^ T lymphocytes following *B. infantis* feeding in the LP (n = 6) is not reversed by citral treatment (n = 5). (**c**) CD4^+^IL-10^+^ T lymphocytes increased within MLN (n = 8), but not the LP (n = 7). LP statistical significance was estimated using the non-parametric Mann-Whitney test, while MLN statistics were determined using the parametric unpaired student t-test.

As previously described, *B. infantis* feeding was associated with increased numbers of regulatory lymphocytes within the mucosa (**Figure 4a**). Foxp3^+^ T lymphocytes were increased within the LP and showed a tendency to be increased for MLN (**Figure 4b**). However, citral did not attenuate the increase in Foxp3^+^ lymphocytes suggesting that mechanisms other than retinoic acid metabolism can be responsible for this effect. IL-10^+^ CD4^+^lymphocytes were significantly increased in the MLN, but not the LP of *B. infantis*-fed animals, which was blocked by citral (**Figure 4c**).

### *B. infantis* attenuation of colitis is retinoic acid-dependent

**Figure 5:**
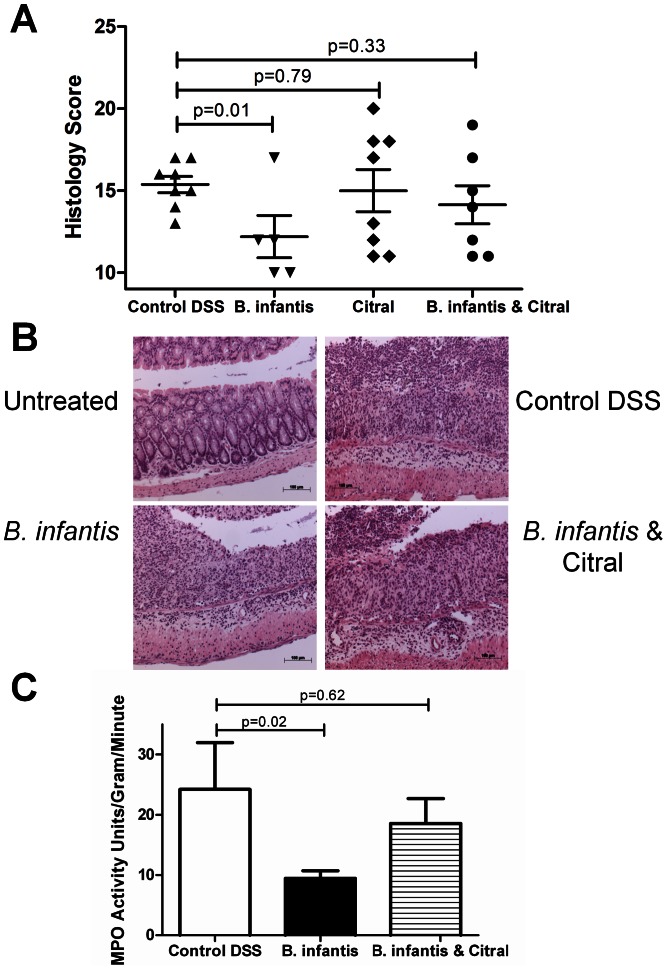
DSS-induced colitis is reduced by *B. infantis* feeding. Four murine study groups were examined – Untreated (no DSS and no *B. infantis*), Control (DSS alone), *B. infantis* (DSS and *B. infantis*), citral (DSS and citral) and *B. infantis* & citral (DSS, *B. infantis* and citral). (**a**) The histopathology inflammatory score was significantly reduced by *B. infantis* feeding (n = 5), but not when citral was co-administered (n = 8). Citral administered with DSS did not increase inflammation in the colon in comparison to DSS alone (n = 7). (**b**) Representative slides of the murine gut are illustrated. (**c**) Colonic myeloperoxidase (MPO) levels were reduced in *B. infantis*-fed mice, which was not observed with *B. infantis* and citral treatment. Statistical significance was determined using non-parametric Mann-Whitney tests.

**Figure 6:**
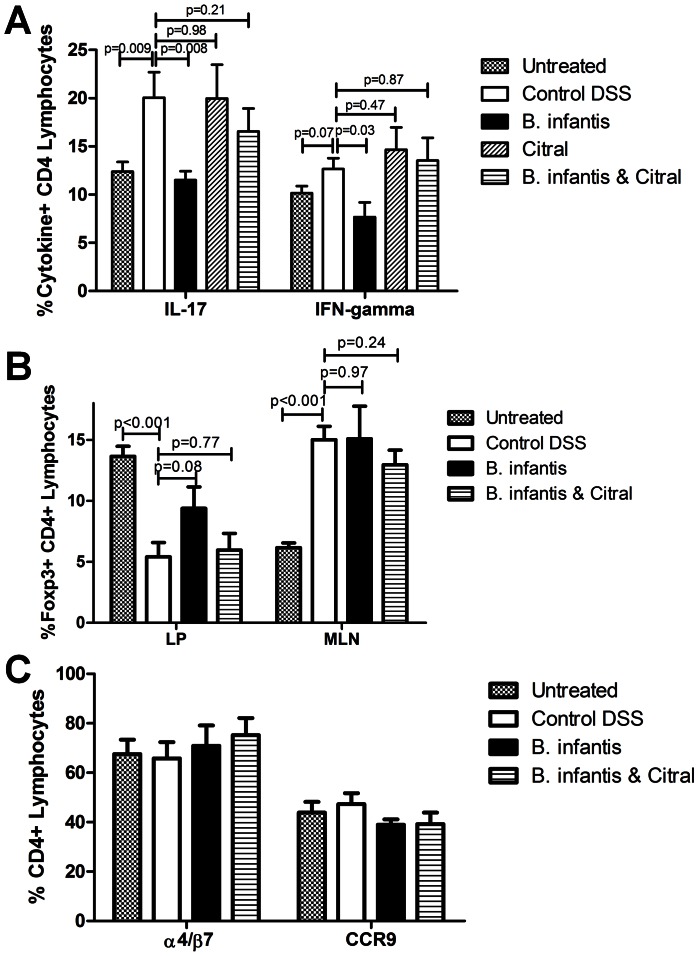
*B. infantis* alters T cell phenotypes within the inflamed LP. (**a**) DSS colitis increases the proportion of IL-17^+^ and IFN-gamma^+^ CD4 T lymphocytes within the LP (n = 8). However, *B. infantis* feeding (n = 6) significantly reduces LP IL-17^+^ and IFN-gamma^+^ subpopulations compared to DSS alone, which was inhibited by citral (n = 7). Both subpopulations were similarly increased in DSS alone and DSS & citral (n = 7). (**b**) CD4^+^Foxp3^+^ lymphocytes were significantly increased in the MLN during DSS colitis (n = 6), with a significant decrease of Foxp3^+^ lymphocytes being observed within the LP (n = 6). *B. infantis* feeding (n = 6) did not alter the increase in Foxp3^+^ cells in the MLN, but partially restored the deficit in Foxp3^+^ cells within the LP (n = 6). (**c**) Expression of the gut homing receptors α4β7 and CCR9 did not significantly change for any of the groups examined. Statistical significance was determined using unpaired student t-tests.

**Figure 7:**
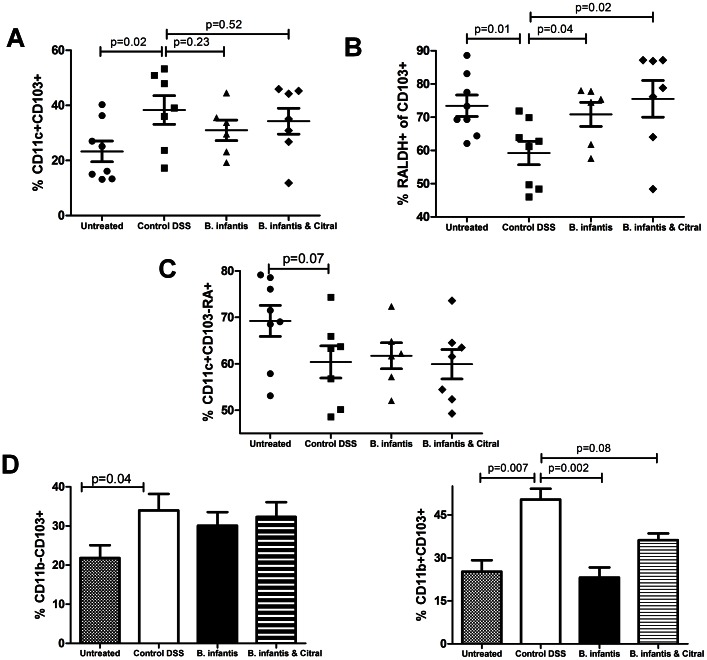
*B. infantis* suppresses the expansion of a pro-inflammatory dendritic cell phenotype within the LP. Untreated (n = 8), DSS colitis (n = 7), DSS and *B. infantis* treated (n = 6) and DSS, *B. infantis* and citral treated animals (n = 7) were compared for dendritic cell subsets within the LP. (**a**) The proportion of CD103^+^ dendritic cells within the LP was increased for all groups with DSS-induced colitis. (**b**) However, the CD103^+^ cells expressing RALDH was significantly reduced in the inflamed LP, which was normalized by *B. infantis* feeding. (**c**) The CD11c^+^CD103^−^RALDH^+^ cell numbers were reduced in the control DSS group and *B. infantis* did not reverse this suppression. (**d**) The increase in CD103^+^ dendritic cells in the inflamed LP primarily consists of CD11b^+^CD103^+^ dendritic cells, while *B. infantis* feeding was associated with an increase in CD11b^−^CD103^+^ dendritic cells. Statistical significance for (a), (b) and (c) was estimated using the non-parametric Mann-Whitney test, while (d) statistics were determined using the parametric unpaired student t-test.

In the DSS model of colitis, *B. infantis* feeding was associated with decreased histopathology (**Figure 5a and 5b**) and decreased MPO levels (**Figure 5c**). Citral treatment blocked the protective effect of *B. infantis* feeding. Citral itself did not impact the severity of DSS-induced colitis (Figure****5a) or LP T_H_1 and T_H_17 subsets (Figure 6a). However, *B. infantis* significantly reduced the numbers of IL-17^+^ and IFN-gamma^+^ lymphocytes within the LP of colitic animals, which was blocked by citral treatment (**Figure 6a**). The proportion of Foxp3^+^ lymphocytes within the MLN increased significantly due to the induction of colitis and *B. infantis* feeding had no effect on the increase in Foxp3^+^ lymphocytes within the MLN (**Figure 6b**). In contrast, DSS-induced colitis was associated with a significantly reduced percentage of Foxp3^+^ lymphocytes within the LP and *B. infantis* feeding partially reversed the drop in Foxp3^+^ lymphocytes within the LP (**Figure 6b**). As described above, *B. infantis* feeding to healthy animals significantly reduced α4β7^+^ and CCR9^+^ lymphocytes within the LP, however *B. infantis* feeding to animals with DSS-induced colitis did not alter α4β7^+^ or CCR9^+^ lymphocyte numbers (**Figure 6c**). Colitis was associated with significantly increased numbers of CD103^+^ dendritic cells within the LP (**Figure 7a**). However, the percentage of CD103^+^ dendritic cells that were RALDH^+^ were significantly reduced in the control DSS group, while *B. infantis* feeding reversed the suppression of RALDH^+^ CD103^+^ dendritic cells (**Figure 7b**). Moreover, the frequency of CD11c^+^ CD103^−^RALDH^+^ cells was reduced in the inflamed LP and *B. infantis* feeding did not affect retinoic acid production by CD103^−^ cells (**Figure 7c**). The upregulation of CD103 in the colitis group was primarily seen on CD11c^+^CD11b^+^ dendritic cells, while the upregulation of CD103 in the *B. infantis* group was within the CD11c^+^CD11b^−^ dendritic cell subpopulation (**Figure 7d**).

## Discussion

In this study, we show that *B. infantis* is sampled by mucosal dendritic cells within the LP and PP, resulting in increased numbers of LP CD103^+^RALDH^+^ dendritic cells with tolerogenic properties. The suppression of T_H_1 and T_H_17 lymphocytes within the LP was observed in the healthy and inflamed gut, which was dependent on retinoic acid metabolism as citral administration blocked this activity. In addition, disease severity was reduced by *B. infantis* feeding during DSS-induced colitis, which was also blocked by citral. Interestingly the elevation in mucosal LP Foxp3 lymphocytes, but not MLN IL-10^+^ lymphocytes, associated with *B. infantis* feeding was RALDH-independent.

LP CD103^+^ dendritic cells are derived from circulating common dendritic cell precursors (not from LP CD103^−^ intermediates), require Flt3 ligand for their development and migrate efficiently to the draining lymph nodes [22], [23]. However, the microbial factors that influence the tolerogenic potency of CD103^+^ dendritic cells within the LP are only beginning to be elucidated. One study using germ-free animals suggested that CD103^+^ dendritic cells within the colon did not require the presence of a microbiota [24]. In contrast, another study recently demonstrated that *Bifidobacterium breve* promoted development of IL-10-producing Tr1 cells in the colon by intestinal CD103^+^ dendritic cells via the TLR2/MyD88-dependent induction of IL-27 and IL-10 [25]. We have also previously demonstrated *in vitro* that *B. infantis*-induced IL-10 secretion by human myeloid dendritic cells was TLR2-dependent [21]. However, Jeon et al did not detect alterations in T_H_17 or T_H_1 populations within the colon following *B. breve* feeding [25]. As they did not measure CD103^+^RALDH activity, direct comparisons with the present study are not possible but it's clear that the *B. infantis*-associated suppression of T_H_17 and T_H_1 cells is dependent on RALDH activity.

Intestinal homeostasis is maintained by regulatory T cell populations consisting of two major CD4^+^ T cell subsets, Foxp3^+^ T_reg_ cells and IL-10-producing Tr1 cells [26]. Site-specific alterations in regulatory lymphocyte subsets are evident in this study. Within the LP, *B. infantis* increased the proportion of Foxp3^+^ lymphocytes, while Tr1 cells were increased only within the MLN. Alternative mechanisms also are required for induction of the two regulatory populations as citral blocked the induction of MLN Tr1 cells, but not the elevation in Foxp3^+^ cells within the LP. Suppression of T_H_1 and T_H_17 lymphocytes within the LP was abrogated by citral confirming that citral administration did have an effect within the LP. Thus, the *B. infantis* induction of Foxp3^+^ lymphocytes within the LP involves mechanisms other than retinoic acid metabolism. Similarly, *B. infantis* reduced the proportion of α4β7 and CCR9 lymphocytes within the LP, which was not dramatically influenced by citral. Retinoic acid has been previously described to upregulate expression of gut homing receptors, which was not observed in our studies. This finding further supports the existence of additional non-RALDH-dependent mechanisms which are induced by *B. infantis* within the LP [27].

Even though RALDH and non-RALDH mechanisms may be required for *B. infantis-*associated immunoregulatory activity within the mucosa, citral blocked the anti-inflammatory effect of *B. infantis* in the DSS colitis model confirming that the induction of retinoic acid metabolism is critical for the *in vivo* protective effects of this microbe. Similar to the findings with healthy mice, *B. infantis* reduced T_H_1 and T_H_17 lymphocytes within the inflamed LP, which was retinoic acid-dependent. In contrast to healthy mice, *B. infantis* did not reduce the number of lymphocytes expressing α4β7 and CCR9 within the inflamed LP. While certain therapeutic approaches have focussed on blocking gut homing receptors for amelioration of colitis, it has also been shown that CCR9 deficiency exacerbates colitis due to impairment of T_reg_ recruitment to the gut [28], [29]. However, our study suggests that *B. infantis* does not alter recruitment of α4β7 or CCR9 positive lymphocytes into the inflamed LP.

During DSS-induced colitis, the relative proportion of CD11b^+^CD103^+^ dendritic cells was increased within the LP, while *B. infantis* feeding was associated with an increase in CD11b^−^CD103^+^ dendritic cells. The LP CD11b^+^CD103^+^ dendritic cell subset has been suggested to possess proinflammatory properties within the inflamed gut [11], [30]. In addition, this dendritic cell population was shown to play key role in T_H_17 cell differentiation *in vitro* [31]. Furthermore, CD11b^+^CD103^+^ dendritic cells express TLR5 at a high level, rapidly respond to flagellin stimulation resulting in IL-23 secretion and are very efficient in presenting antigens to CD4+ lymphocytes [12], [32], [33]. Our data suggests that *B. infantis*, even within an inflamed microenvironment, continues to induce regulatory CD11b^−^CD103^+^RALDH^+^ dendritic cells within the LP and suppresses the increase in the proinflammatory CD11b^+^CD103^+^ dendritic cell population. Interestingly, the suppression of T_H_17 cells within the inflamed LP of *B. infantis*-fed mice correlates with the suppression of the CD11b^+^CD103^+^ dendritic cell subset. Further investigation is required to determine if there is a direct connection between *B. infantis* associated suppression of CD11b^+^CD103^+^ dendritic cells and T_H_17 polarisation.

Within the mucosa, dendritic cells are integral to promoting oral tolerance and preventing pathological immune responses to harmless antigens. Dendritic cells use signals derived from their local environment to shape regulatory and low-level immune responses to the commensal microbiota, which controls the microbiota without causing pathology. The breakdown in dendritic cell regulatory networks is associated with aberrant inflammatory activity within the gut and therapeutic strategies aimed at re-establishing dendritic cell tolerogenic tone would be of benefit to IBD, IBS and food allergy patients. One such strategy is the deliberate manipulation of CD103^+^RALDH^+^ dendritic cells by microbes or microbial components in combination with dietary supplementation with vitamin A. The murine data presented in this report strongly support the further evaluation of these strategies in human clinical studies.

## Supporting Information

Figure S1 ***B. infantis*** **is bound at a low frequency by CD11c^+^MHCII^+^CD103^−^ dendritic cells.** Flow cytometric analysis of CD11c^+^MHCII^+^CD103^−^ dendritic cells from the mucosa demonstrated that approximately 10% of CD103^−^ dendritic cells bound *B. infantis*.
(PPT)
