# Supplementary figures and images for: Immunomodulation by Bifidobacterium infantis 35624 in the Murine Lamina Propria Requires Retinoic Acid-Dependent and Independent Mechanisms
Source: PLoS One. 2013 May 21;8(5):e62617. doi: 10.1371/journal.pone.0062617 (PMC3660574; doi:10.1371/journal.pone.0062617)

## Slide 1
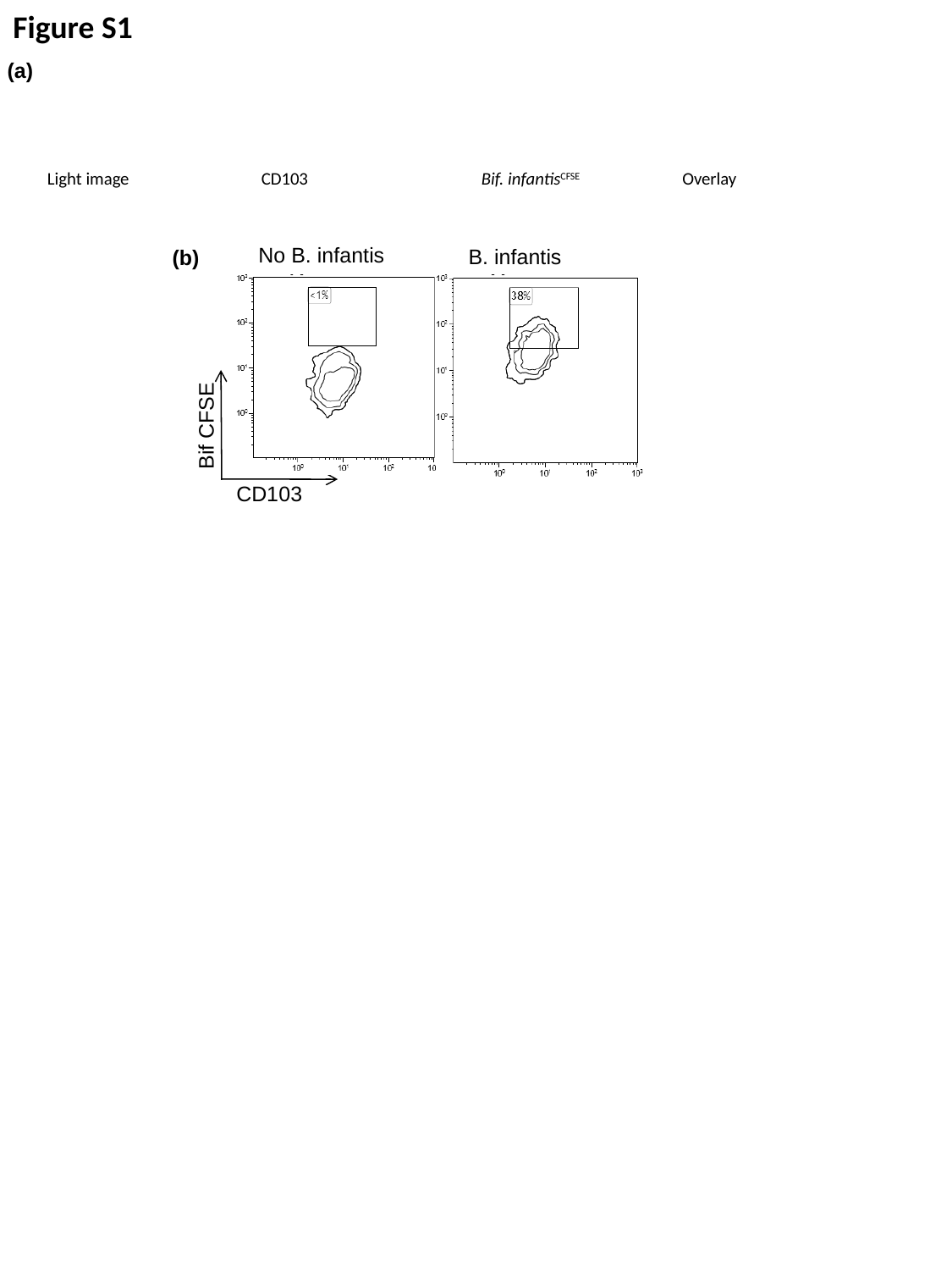

Figure S1
(a)
Light image CD103 Bif. infantisCFSE Overlay
No B. infantis
B. infantis
Bif CFSE
CD103
(b)

Supplement: Figure S1 — B. infantis is bound at a low frequency by CD11c+MHCII+CD103− dendritic cells. Flow cytometric analysis of CD11c+MHCII+CD103− dendritic cells from the mucosa demonstrated that approximately 10% of CD103− dendritic cells bound B. infantis. (PPT) [file pone.0062617.s001.ppt]
